# Two decades of rice research in Indonesia and the Philippines: A systematic review and research agenda for the social sciences

**DOI:** 10.1057/s41599-022-01394-z

**Published:** 2022-10-14

**Authors:** Ginbert P. Cuaton, Laurence L. Delina

**Affiliations:** The Hong Kong University of Science and Technology, Hong Kong SAR, China

**Keywords:** Development studies, Environmental studies

## Abstract

While rice studies are abundant, they usually focus on macro-level rice production and yield data, genetic diversity, cultivar varieties, and agrotechnological innovations. Moreover, many of these studies are either region-wide or concentrated on countries in the Global North. Collecting, synthesizing, and analyzing the different themes and topic areas in rice research since the beginning of the 21st century, especially in the Global South, remain unaddressed areas. This study contributes to filling these research lacunae by systematically reviewing 2243 rice-related articles cumulatively written by more than 6000 authors and published in over 900 scientific journals. Using the PRISMA 2020 guidelines, this study screened and retrieved articles published from 2001 to 2021 on the various topics and questions surrounding rice research in Indonesia and the Philippines—two rice-producing and -consuming, as well as emerging economies in Southeast Asia. Using a combination of bibliometrics and quantitative content analysis, this paper discusses the productive, relevant, and influential rice scholars; key institutions, including affiliations, countries, and funders; important articles and journals; and knowledge hotspots in these two countries. It also discusses the contributions of the social sciences, highlights key gaps, and provides a research agenda across six interdisciplinary areas for future studies. This paper mainly argues that an interdisciplinary and comparative inquiry of potentially novel topic areas and research questions could deepen and widen scholarly interests beyond conventional natural science-informed rice research in Indonesia and the Philippines. Finally, this paper serves other researchers in their review of other crops in broader global agriculture.

## Introduction

Rice feeds the majority of the world’s population and employs millions, especially in developing countries in the Global South (Muthayya et al., 2014). Rice consumption has increased globally over the last decade. Statista data show that, in the cropping year 2020/2021, the world population consumed about 504.3 million metric tons of rice, increasing from 437.18 million metric tons in 2008/2009 (Shabandeh, 2021). These data highlight the crop’s global contribution and importance, especially in realizing the Sustainable Development Goals (SDGs), the blueprint for global prosperity (Gil et al., 2019). The SDGs call for systems transformation, including in agriculture, guided by the principles of sustainability and equity, driven by the leave-no-one-behind aphorism, to address the root causes of perennial poverty and chronic hunger.

Pathologist M. B. Waite (1915) pointed out that the apparent indicator of progress in modern agriculture is the application of scientific research and the subsequent modification and improvement of farming systems based on those research. For example, the Green Revolution resulted in increased agricultural production in developing countries due to the transfer of agrotechnological innovations from countries in the Global North to countries in the Global South. Although, we acknowledge that this project came with a cost (Glaeser, 2010; Pielke and Linnér, 2019; Pingali, 2012).

Regional rice studies have proliferated in Europe (Ferrero and Nguyen, 2004; Kraehmer et al., 2017), the Americas (Singh et al., 2017), Africa (Zenna et al., 2017), the Asia Pacific (Papademetriou et al., 2000), and South Asia (John and Fielding, 2014). Country studies on rice production have also emerged in Australia (Bajwa and Chauhan, 2017), China (Peng et al., 2009), and India (Mahajan et al., 2017). Scholars have also systematically reviewed rice’s phytochemical and therapeutic potentials (Sen et al., 2020), quality improvements (Prom-u-thai and Rerkasem, 2020), and its role in alleviating the effects of chronic diseases and malnutrition (Dipti et al., 2012).

These extant studies, however, are limited on at least three fronts. First, their foci were on rice production, yield, and operational practices and challenges at the macro level. Second, there have been zero attempts at synthesizing this corpus since the 21st century. Third, there are also no attempts at examining the various rice research areas that scholars, institutions, and countries need to focus on, especially in developing country contexts, and their nexuses with the social sciences. This paper addresses these gaps by unpacking and synthesizing multiple rice studies conducted in the emerging Southeast Asian economies of Indonesia and the Philippines from 2001 to 2021. A focus on these developing countries matters since they are home to over 35 million rice farmers (IRRI, 2013).

We conducted our review from the Scopus database, using a combination of bibliometric and quantitative content analyses. Section “Results and discussions” reports our results, where we discuss (1) the most relevant and influential rice scholars and their collaboration networks; (2) the most rice research productive institutions, including author affiliations, their countries, and their research funders; and (3) the most significant articles and journals in rice research. This section also identifies 11 topic areas belonging to four major themes of importance for rice research in the two countries. Section “Contributions from and research agenda for the social sciences” provides a research agenda, where we identify and discuss the contributions of our review in terms of future work. Despite the preponderance of rice research in the last two decades and more in Indonesia and the Philippines, contributions from the social sciences remain marginal. Thus, in the section “Conclusion”, we conclude that emphasis is needed on expanding and maximizing the contributions of social scientists given the many opportunities available, especially for conducting interdisciplinary and comparative rice research in these Southeast Asian countries.

## Review methods and analytical approach

We used bibliometric and quantitative content analyses to systematically categorize and analyze more than two decades of academic literature on rice in Indonesia and the Philippines. Bibliometric methods, also known as bibliometrics, have grown to be influential in evaluating various research fields and topic areas. Bibliometrics mushroomed because of the increasing availability of online databases and new or improved analysis software (Dominko and Verbič, 2019). Bibliometrics quantitatively and statistically analyze research articles using their bibliographic data, such as authors, affiliations, funders, abstracts, titles, and keywords. These data are analyzed to identify and assess the development, maturity, research hotspots, knowledge gaps, and research trends (Aria and Cuccurullo, 2017). For example, bibliometrics have been used in reviewing hydrological modeling methods (Addor and Melsen, 2019), business and public administration (Cuccurullo et al., 2016), and animals’ cognition and behavior (Aria et al., 2021).

This review article used *bibliometrix*, a machine-assisted program that offers multiple options and flexibility to map the literature comprehensively (Aria and Cuccurullo, 2017). We run this program using R Studio version 4.1.2 (2021-11-01; “Bird Hippie”) for its source code readability, understandability, and easy-to-do computer programming (Cuaton et al., 2021). We used bibliometrix in three critical analytical phases: (a) importing and converting data to R format, (b) identifying our dataset’s collaboration networks and intellectual and conceptual structures, and (c) processing, presenting, and analyzing our dataset. Bibliometrix, however, is unable to produce specific data that we want to highlight in this paper; examples of these are our coding criteria on interdisciplinarity and author gender, where such information was not captured in the articles’ bibliographic data in Scopus. We addressed these issues by conducting a quantitative content analysis (QCA) of our dataset. QCA is a method to record, categorize, and analyze textual, visual, or aural materials (Coe and Scacco, 2017). QCA has been applied in other reviews, such as in energy research development in the social sciences (Sovacool, 2014), the concepts of energy justice (Jenkins et al., 2021), and in examining agricultural issues in Botswana (Oladele and Boago, 2011) and Bangladesh (Khatun et al., 2021).

### Search strategies

We constructed our dataset from the Scopus database, which we accessed via our institution’s online library on 14 November 2021. Scopus is a scientific database established in 2004 and owned by Elsevier Ltd. (Elsevier, 2021). We excluded other databases, such as Google Scholar, ScienceDirect, Web of Science, and EBSCO, suggesting one potential bias in our review (Waltman, 2016; Zupic and Čater, 2015). Our decision to exclusively use Scopus arises from two main reasons. First, the database has broader coverage than others, including the abovementioned (Falagas et al., 2008). Scopus includes new and emerging journals published in developing countries like Indonesia and the Philippines, our focus countries. Second, Scopus has a user-friendly interface and its search options allow researchers to flexibly explore its universe of indexed articles based on authors, institutions, titles, abstracts, keywords, and references (Donthu et al., 2021).

**Fig. 1:**
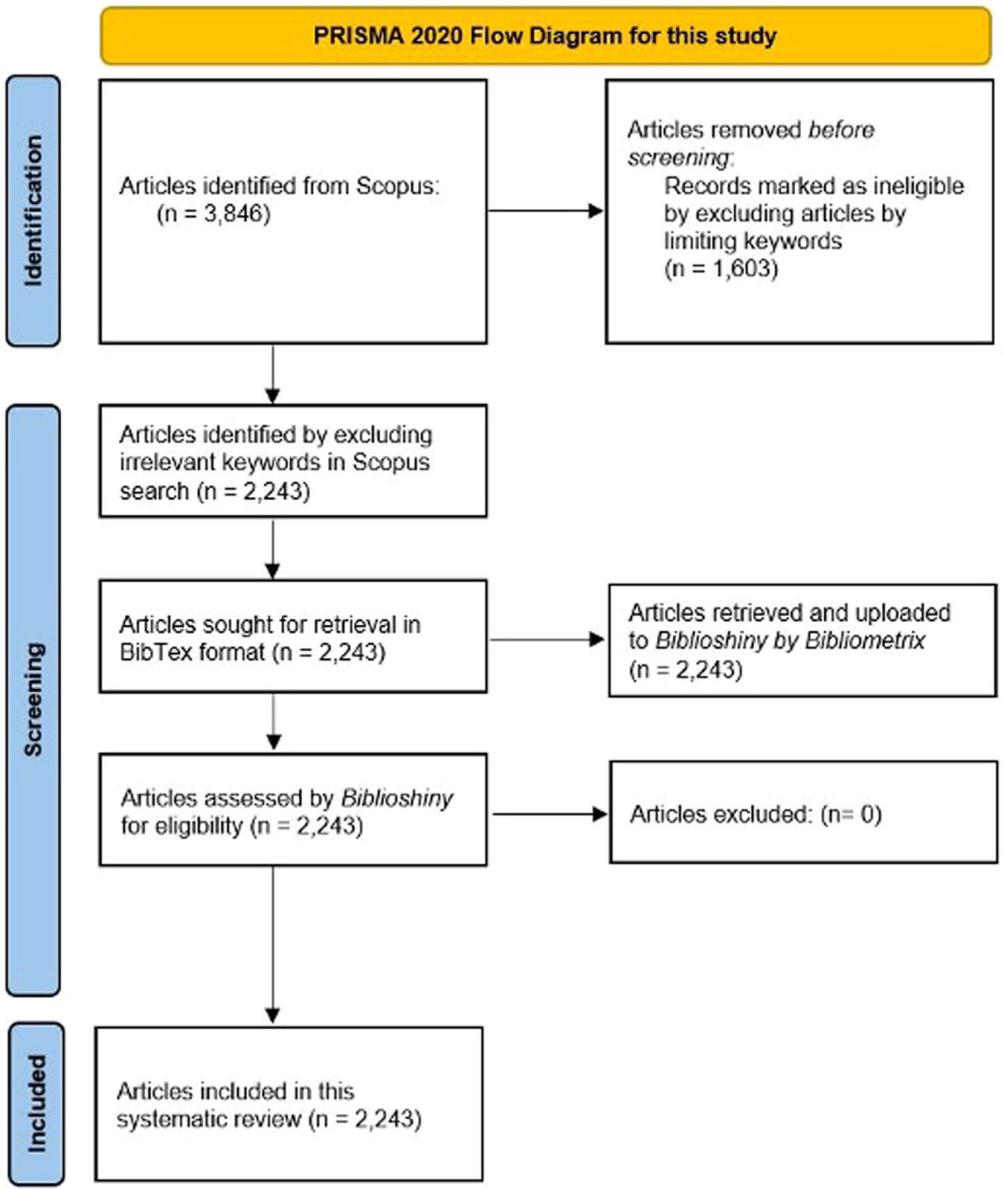
The PRISMA 2020 flow diagram for this systematic review. Our initial result of 3846 documents (results as of 14 November 2021) was filtered by including only peer-reviewed, full-text English articles on rice, resulting in 2243 eligible documents.

We followed the PRISMA 2020 Guideline (Preferred Reporting Items for Systematic reviews and Meta-Analyses) (Page et al., 2021) in our search for potential rice-related studies in Indonesia and the Philippines (see Fig. 1). We used the initial search string: “rice” AND “Indonesia*” OR “Philippine*” (asterisk or “*” was used as a wildcard search strategy) and limited the year coverage from 2001 to 2021. Our first round of searches resulted in 3846 documents (results as of 14 November 2021). We filtered these documents by including only peer-reviewed, full-text English articles on rice. We did not include any documents from the grey literature (e.g., news items, press releases, government or corporate reports), and other document types indexed in Scopus such as reviews, books, conference papers, errata, comments, editorials, and short reports.

We also excluded articles with irrelevant keywords by using the following combined queries:

**Table:** 

(TITLE-ABS-KEY (rice) AND TITLE-ABS-KEY (Indonesia*) OR TITLE-ABS-KEY (Philippine*)) AND PUBYEAR > 2000 AND PUBYEAR < 2022 AND (LIMIT-TO (DOCTYPE, “ar”)) AND (EXCLUDE (EXACTKEYWORD, “*Zea Mays*”) OR EXCLUDE (EXACTKEYWORD, “Maize”) OR EXCLUDE (EXACTKEYWORD, “Viet Nam”) OR EXCLUDE (EXACTKEYWORD, “India”) OR EXCLUDE (EXACTKEYWORD, “Thailand”)) AND (EXCLUDE (EXACTKEYWORD, “Cacao”) OR EXCLUDE (EXACTKEYWORD, “Cacao Shell”) OR EXCLUDE (EXACTKEYWORD, “Cambodia”)).	

This resulted in 2243 eligible documents. We downloaded these documents as raw files in BibTex format and imported them to *Biblioshiny*, a web interface in Bibliometrix, where they were further filtered. Our verified final dataset comprises 2243 full-text English articles cumulatively written by 6893 authors and published across 909 journals (see Table 1).

**Table 1:** Dataset snapshot.

Main information		Document contents	
Timespan	2001–2021	Keywords Plus	6858
Journals	909	Author’s Keywords	6482
Documents	2243	*Authors collaboration*	
Average citations per doc	13.04	Single-authored documents	194
References	79,955	Authors per Document	3.07
*Authors*		Co-Authors per Documents	4.35
Authors (cumulative)	6893	Collaboration Index	3.28

## Structure and analytical approach

### Authors

We examined the authors’ profiles based on their gender, relevance in the study, and global impact. For gender, we coded them into ‘man,’ ‘woman,’ and ‘undetermined’ because some did not put enough information that helps in gender identification. We identified their gender by counter-checking their Scopus profiles to their verified accounts in Google Scholar, ResearchGate, Publons/Web of Science, or institutional profiles. We measured the authors’ relevance and impact against their (a) productivity, (b) citations, and (c) H-indices. We acknowledge, however, that some Filipino and Indonesian scholars, whose papers may not be indexed in Scopus, could also be prolific based on different parameters, but we excluded them. We proceeded to map the collaboration networks of these authors to identify “who works with whom on what.” A collaboration network illustrates nodes (circle shape) as authors and links (connecting lines) as co-authorships (Glänzel and Schubert, 2005).

### Institutions, countries, funders

Following Sovacool (2014), we categorized the authors’ institutions into four: (1) *University and research* included authors who are researchers, instructors/lecturers/professors, other academic faculty from various non-university research think tanks, institutes, and national and local research centers; (2) *Government* consisted country or state departments, bureaus, ministries, and other government regulatory bodies; (3) *Interest groups and NGOs* included intergovernmental bodies, such as the United Nations Food and Agriculture Office (FAO) and international organizations like the International Rice Research Institute (IRRI) and Oxfam; and (4) *Banking and finance* encompassed players from the finance sector, including multilateral development banks such as the Asian Development Bank (ADB), World Bank, and the International Fund for Agricultural Development (IFAD). After coding and categorizing, we analyzed the authors’ institutional collaboration networks.

We identified the country’s productivity and coded them by global region based on their geographical location: (a) Asia, (b) Australia, New Zealand, and South Pacific, (c) Europe, (d) North America, (e) South America, and (f) Africa. We did this to show how various countries have been researching rice in Indonesia and the Philippines since the 21st century.

We then constructed a country collaboration map as a visual macro-representation of countries working together on rice research using these data. Bibliometrix, however, measured the country’s productivity based on the corresponding authors’ affiliations. We, therefore, noted two critical points here. First, many corresponding authors may have multiple institutional affiliations. For example, one corresponding author may belong to more than two affiliations (e.g., a corresponding Filipino author may have concurrent institutional affiliations in Japan, Australia, and New Zealand). Second, the corresponding authors may not necessarily be nationals of that country. Note that the unit of analysis is based on the corresponding authors’ institutional affiliations at the time of publication and not on their country/ies of citizenship or nationality. Despite these, our findings still provide insight into the macro-level productivity of countries conducting rice research in Indonesia and the Philippines.

We analyzed the funders using Scopus’ in-house Analytics Tool and determined their relevance based on the number of articles mentioning them in the Funding source or Acknowledgment section in the paper. We categorized the funders into six: (1) government (e.g., ministries, departments, or regulatory agencies), (2) research (e.g., research councils, research centers, and national academies), (3) foundations and non-government organizations (NGOs), (4) universities, (5) private companies and corporations, and (6) intergovernmental organizations/IGOs, including multilateral development banks.

### Articles and journals

In terms of interdisciplinarity, we coded the articles as (a) interdisciplinary, (b) disciplinary, or (c) unidentified by using the authors’ department or division affiliation/s as a proxy to determine their disciplinary training. We coded an article as interdisciplinary if it belonged to any of the three criteria: (1) it had an author that had training or belonged to a department/division in at least two conventional disciplines (e.g., agriculture, anthropology, sociology, biology); (2) it had an author that had a self-identified interdisciplinary department (e.g., interdisciplinary division, sustainability, agriculture economics, etc.); or (3) it had at least two authors with different disciplinary training or expertise (e.g., business and economics; crop science and political science, etc.). We coded an article as disciplinary if its author/s had only belonged to one conventional department/division affiliation (e.g., Division of Agriculture, Department of Economics, Division of Environmental Science, etc.). On the other hand, we coded an article as undetermined when the authors had only indicated the name of their institutions or did not indicate their departmental or division affiliations (e.g., only the University of the Philippines, IRRI, Universitas Gadja Mada, etc.).

We examined the articles based on their local relevance and global influence. Bibliometrix measured the articles’ relevance based on their “local citations” or citations received from the 2243 articles of our sample dataset. We did this to determine which papers are considered relevant by authors studying various areas of rice research in Indonesia and the Philippines. Global influence is measured based on the articles’ citations from the global research community or other scientific works beyond our sample dataset. We also conducted a co-citation analysis of the cited references. Co-citation is the frequency by which articles cite together two or more articles relevant to the topic areas of inquiry (Aria and Cuccurullo, 2017). Bibliometrix had identified some co-cited articles published before our timeline of interest (i.e., pre-2001) which provide scholars with a more profound understanding of rice research in the two countries.

On the other hand, Bibliometrix identified the most relevant journals based on the number of papers the journals had published and the local citations of the articles. These data guide readers and researchers on which journals to look for on rice studies in Indonesia and the Philippines.

### Knowledge hotspots

Bibliometrix creates a thematic map that allows researchers to identify which study areas have been adequately explored and which areas need further investigation or re-investigation to identify knowledge hotspots and research gaps (Aria and Cuccurullo, 2017). Della Corte et al. (2019, pp. 5–6) discussed the major themes in Bibliometrix in the following:

“Themes in the lower-right quadrant are the *Basic Themes*, characterized by high centrality and low density. These themes are considered essential for a research field and concerned with general topics across different research areas.

Themes in the upper-right quadrant are the *Motor Themes*, characterized by high centrality and density. Motor themes are considered developed and essential for the research field.

Themes in the upper-left quadrant are the highly developed and isolated themes or *Niche Themes*. They have well-developed internal links (high density) but unimportant external links, which could be interpreted as having limited importance for the field (low centrality).

Themes in the lower-left quadrant are known as *Emerging or Declining Themes*. They have low centrality and density, making them weakly developed and marginal.”

### Contributions from and research agenda for the social sciences

As interdisciplinary environmental and social scientists, we also focused our review on the social studies of rice in the two countries. This section highlighted the gaps between the natural and the social sciences in rice research and advanced a research agenda for interdisciplinary and comparative social scientists.

### Limitations

As in any systematic review, we acknowledge certain limitations to our work. We discuss four of these.

First, to keep a certain level of reliability, we focused only on peer-reviewed full-length research articles written in the English language and indexed in the Scopus database. Therefore, we may have excluded some relevant articles, including those written in Filipino, Indonesian, and other local or indigenous languages and published in local or international journals but are not indexed in Scopus. Our review also excluded conference papers, commentaries, book reviews, book chapters, conference reviews, data papers, errata, letters, notes, and non-academic publications like policy briefings, reports, and white papers.

Second, in our quantitative content analysis, we acknowledge the highly cis-heteronormative approach we used in coding the author’s gender as “man” or “woman.” We identified these genders from the names and pictures of the authors in their verified Scopus, Publons/ Web of Science, and institutional profiles. It is not our deliberate intention to neglect the varying genders of researchers and scientists beyond the traditional binary of man or woman.

Third, we recognize that our analysis cannot directly identify how much each funder provided as the unit of analysis in bibliometrix may depend on how prolific researchers were in publishing articles despite smaller funds. For instance, one research project supported by Funder A with US$1 million may have published only one article based on their project design or the funder's requirement. Since the authors published only one paper from this project, the data could show that Funder A only funded one research. Another research project, supported by Funder B, with only US$300,000 in funding, may have published more than five papers; therefore, more articles counted as funded by Funder B. This issue is not within the scope of our review.

Lastly, it should be noted that the future research works we discussed were highly influenced by our research interests and the general overview of the literature, and thus neither intend to cover nor aim to discuss the entire research topics that other scholars could study.

Despite these limitations, we strongly argue that our review provided relevant insights and proposed potentially novel topic areas and research questions for other scholars to explore, especially social scientists, in deepening and widening rice research in Indonesia and the Philippines. To end, we hope that researchers heed our call to conduct more interdisciplinary and comparative rice-related studies in these two emerging Southeast Asian countries.

## Results and discussions

**Fig. 2:**
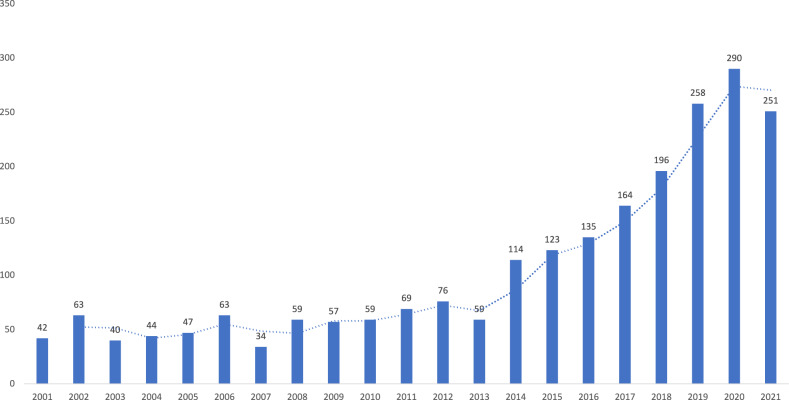
Average annual publications of rice research in Indonesia and the Philippines from 2001 to 2021. The average number of annual publications on rice research in Indonesia and the Philippines from 2001 to 2013 was only 57 papers but elevated to hundreds beginning in 2014.

Our dataset comprises 2243 peer-reviewed journal articles cumulatively written by 6893 authors who cited around 80,000 cumulative references. The average annual publications from 2001 to 2013 were only 57 papers but elevated to hundreds beginning in 2014 (Fig. 2).

### Authors

Of the 159 authors, one had a duplicate profile; thus, we identified 158 authors publishing on rice studies; the majority (66%) are men. The top 50 most prolific scholars produced a little over 25% (567 articles) of the total articles. Australian ecologist Finbarr Horgan topped this list (*n* = 21), followed by Bas Bouman and Grant Singleton—each with 20 articles. The top 10 authors with the highest number of publications have affiliations with the IRRI, the University of the Philippines, the University of Gadjah Mada, and the Philippine Rice Research Institute (PhilRice). For the full list of prolific scholars with at least 10 articles published, see Supplementary Table 1.

In terms of the authors with the most local citations, although Finbarr Horgan has the most documents, Johan Iskandar (*n* = 36 citations) from the Universitas Padjadjaran, who studies rice genetic diversity, is the most cited. Local citations refer to the citations received by authors from our sample dataset of 2243 articles. Muhidin Muhidin from the Universitas Halu Oleo and Ruhyat Partasasmita from the Universitas Padjadjaran, followed him with 30 and 28 local citations, respectively. Common to these three authors are their Biology background/expertise and interest in rice genetic diversity. To check the top 20 most locally cited scholars, refer to Supplementary Table 2.

The H-index is the author-level measure of publications’ productivity and citation impacts (Hirsch, 2005). Bas Bouman (H-index = 18) leads the top 10 scholars among rice-related researchers in Indonesia and the Philippines. Yoshimichi Fukuta (H index = 13) and Shaobing Peng (H index = 13) followed him. These three authors are affiliated with or have collaborated with the IRRI. To check the top 10 scholars with the highest H-indices, refer to Supplementary Table 3.

**Fig. 3:**
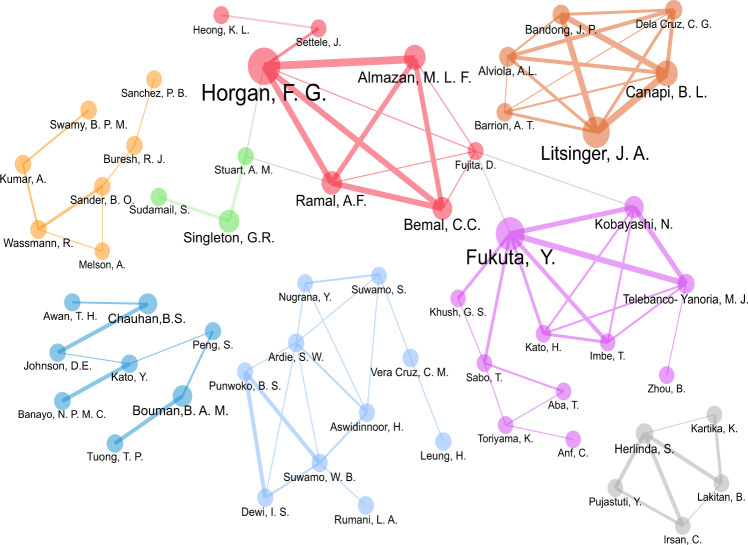
Author collaboration networks, *n* = 80 authors. The authors’ collaboration networks show eight major clusters of rice research in Indonesia and the Philippines.

Figure 3 reveals the top 80 authors who collaborate across eight major clusters of rice research. The Red cluster shows Finbarr Horgan as the most prominent author with at least four significant collaborators in pest management, specifically on rice stemborers (Horgan et al., 2021), anthropods’ biodiversity in tropical rice ecosystems (Horgan et al., 2019), and virulence adaptations of rice leafhoppers (Horgan et al., 2018). In the Purple Cluster, Yoshimichi Fukuta has multiple publications with at least six collaborators in the study of rice blast (Ebitani et al., 2011; Kadeawi et al., 2021; Mizobuchi et al., 2014). In the Brown cluster, Bernard Canapi from the IRRI has collaborated with at least five scholars in the study of rice insect pest management (Cabasan et al., 2019; Halwart et al., 2014; Litsinger et al., 2011), farmers’ preference for rice traits (Laborte et al., 2015), and the drivers and consequences of genetic erosion in traditional rice agroecosystems in the Philippines (Zapico et al., 2020). The Gray cluster shows that Siti Herlinda has collaborated with at least four scholars to study anthropods in freshwater swamp rice fields (Hanif et al., 2020; Herlinda et al., 2020) and the benefits of biochar on rice growth and yield (Lakitan et al., 2018).

### Institutions

#### Author affiliations

**Fig. 4:**
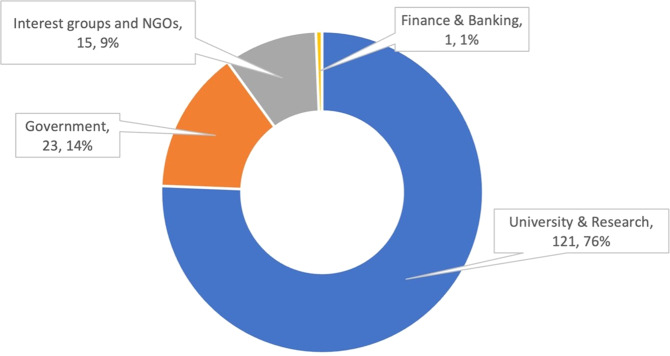
Institutional distribution of rice researchers in Indonesia and the Philippines, *n* = 160 institutions. The majority of rice researchers in Indonesia and the Philippines have affiliations with “University and research”.

**Fig. 5:**
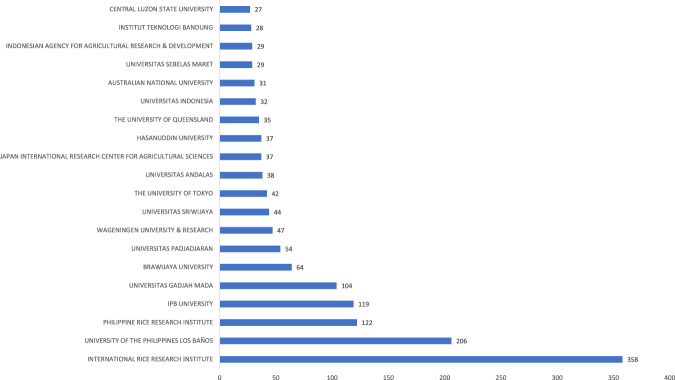
Affiliations of rice researchers in Indonesia and the Philippines, *n* = 20 affiliations. The top 5 most productive institutions in terms of rice research in Indonesia and the Philippines are the IRRI, the University of the Philippines System, the PhilRice, the Institute Pertanian Bogor/IPB University, and the University of Gadja Mada.

In terms of institutional types, Fig. 4 shows that most rice researchers in Indonesia and the Philippines have affiliations with “University and research.” Figure 5 shows the top 20 institutions in terms of research productivity led by the IRRI, the University of the Philippines System, the PhilRice, the Institute Pertanian Bogor/IPB University, and the University of Gadja Mada. These 20 institutions produced 66% of the articles in our dataset.

Scholars affiliated with the IRRI have written the most papers (at least 19% or 358 articles) in our dataset. The range of topics covers both regional and country studies. Some regional examples include the drivers of consumer demand for packaged rice and rice fragrance in South and Southeast Asia (Bairagi et al., 2020; Bairagi, Gustafson et al., 2021). Country studies, for example, include an investigation of rice farming in Central Java, Indonesia (Connor et al., 2021), the cultural significance of heirloom rice in Ifugao in the Philippines (Bairagi, Custodio et al., 2021), and the distributional impacts of the 2019 Philippine rice tariffication policy (Balié and Valera, 2020).

The University of the Philippines System, with rice scholars affiliated with their campuses in Los Baños, Diliman, Mindanao, and Manila, produced the next largest number of papers (more than 200 or 10%) on topics about rice pests and parasites (Horgan et al., 2019, 2021; Vu et al., 2018), weed control (Awan et al., 2014, 2015; Fabro and Varca, 2012), and climate change impacts on rice farming (Alejo and Ella, 2019; Ducusin et al., 2019; Gata et al., 2020). Social studies of rice conducted by the University of the Philippines researchers include indigenous knowledge on climate risk management (Ruzol et al., 2020, 2021), management options in extreme weather events (Lopez and Mendoza, 2004), agroecosystem change (Aguilar et al., 2021; Neyra-Cabatac et al., 2012), and the development and change over time of rice production landscapes (Santiago and Buot, 2018; Tekken et al., 2017).

PhilRice, a government-owned corporation under the Department of Agriculture (Official Gazette of the Philippines, 2021), is the third most prolific rice research-producing institution (122 papers) on topics ranging from nematodes or rice worms (Gergon et al., 2001, 2002) and arthropods (invertebrates found in rice paddies) (Dominik et al., 2018), hybrid rice (Perez et al., 2008; Xu et al., 2002), alternate wetting-and-drying technology (Lampayan et al., 2015; Palis et al., 2017), and community development strategies on rice productions (Romanillos et al., 2016).

The IPB University, a public agrarian university in Bogor, Indonesia, investigates rice productivity and sustainability (Arif et al., 2012; Mucharam et al., 2020; Setiawan et al., 2013), irrigation (Nugroho et al., 2018; Panuju et al., 2013), extreme weather events such as drought (Dulbari et al., 2021), floods (Wakabayashi et al., 2021), and emerging social issues such as food security (Putra et al., 2020), land-use change (Chrisendo et al., 2020; Munajati et al., 2021), and sustainability (Mizuno et al., 2013). This university has 23 research centers, including those which focus on environmental research; agricultural and village development; engineering applications in tropical agriculture; Southeast Asian food and agriculture; and agrarian studies.

Universitas Gadja Maja in Yogyakarta, Indonesia, hosts 21 research centers, including its Agrotechnology Innovation Centre. It carries out research incubation and development activities, product commercialization, and integration of agriculture, animal husbandry, energy, and natural resources into a sustainable Science Techno Park. Some of their published studies focused on drought-tolerant rice cultivars (Salsinha et al., 2020, 2021; Trijatmiko et al., 2014), farmers’ technical efficiency (Mulyani et al., 2020; Widyantari et al., 2018, 2019), systems of rice intensification (Arif et al., 2015; Syahrawati et al., 2018), and climate change adaptation (Ansari et al., 2021).

**Fig. 6:**
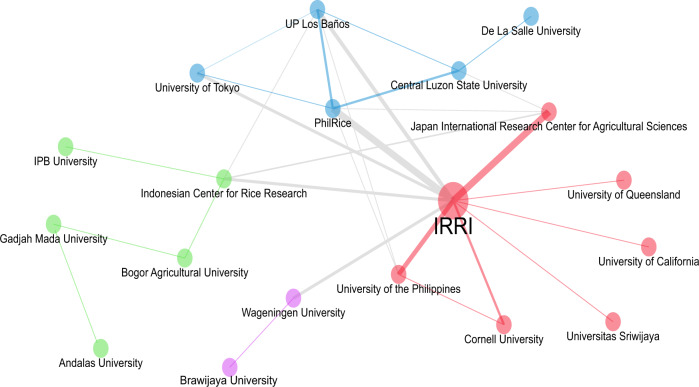
Institutional collaboration networks of rice researchers in Indonesia and the Philippines, *n* = 30 institutions. The IRRI, as an international organization focused on many aspects of rice, is not surprising to have the greatest number of institutional collaborators (*n* = 11 institutions).

In terms of institutional collaboration, the IRRI tops the list with at least eleven collaborators (Fig. 6), including the Japan International Center for Agricultural Sciences, the PhilRice, the University of the Philippines System, and the Indonesian Center for Rice Research.

#### Countries

**Fig. 7:**
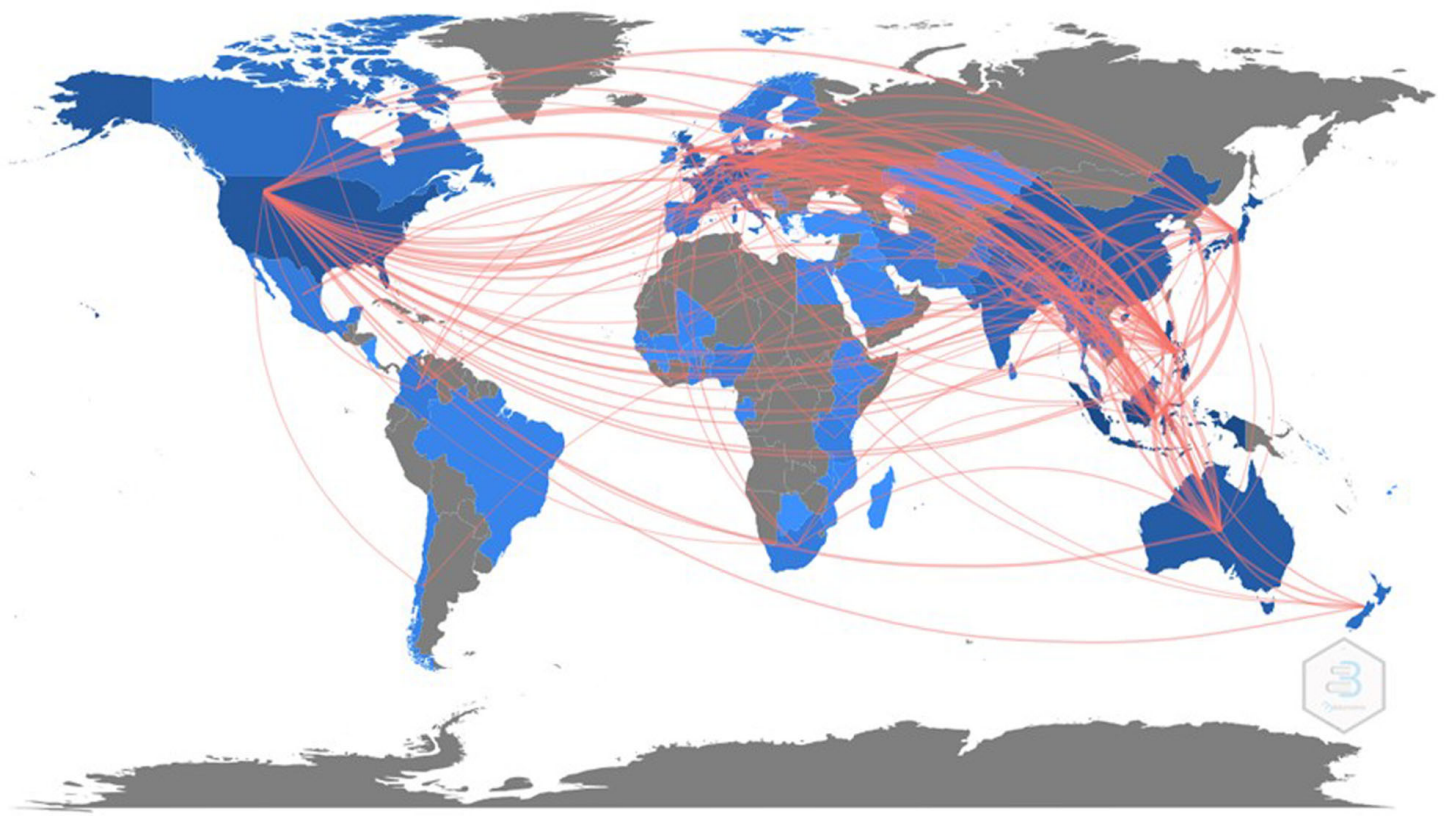
Country collaboration networks of rice-related projects in Indonesia and the Philippines since 2001. A macro-level picture of how countries have collaborated on rice-related projects in Indonesia and the Philippines since 2001. It suggests that rice research in both countries has benefited from international partnerships.

Rice studies’ authors are from at least 79 countries; the majority of them are working in Asia (79%), followed by Europe (13%) and North America (9%). At least 90% of rice scholars are in Indonesia, and more than 51% have affiliations in the Philippines, followed by Japan, the USA, and China. For the list of the top 20 most productive countries researching rice in Indonesia and the Philippines, see Supplementary Table 4. Figure 7 shows a macro-level picture of how countries have collaborated on rice-related projects in Indonesia and the Philippines since 2001, suggesting that rice research in both countries has benefited from international partnerships.

#### Funders

**Fig. 8:**
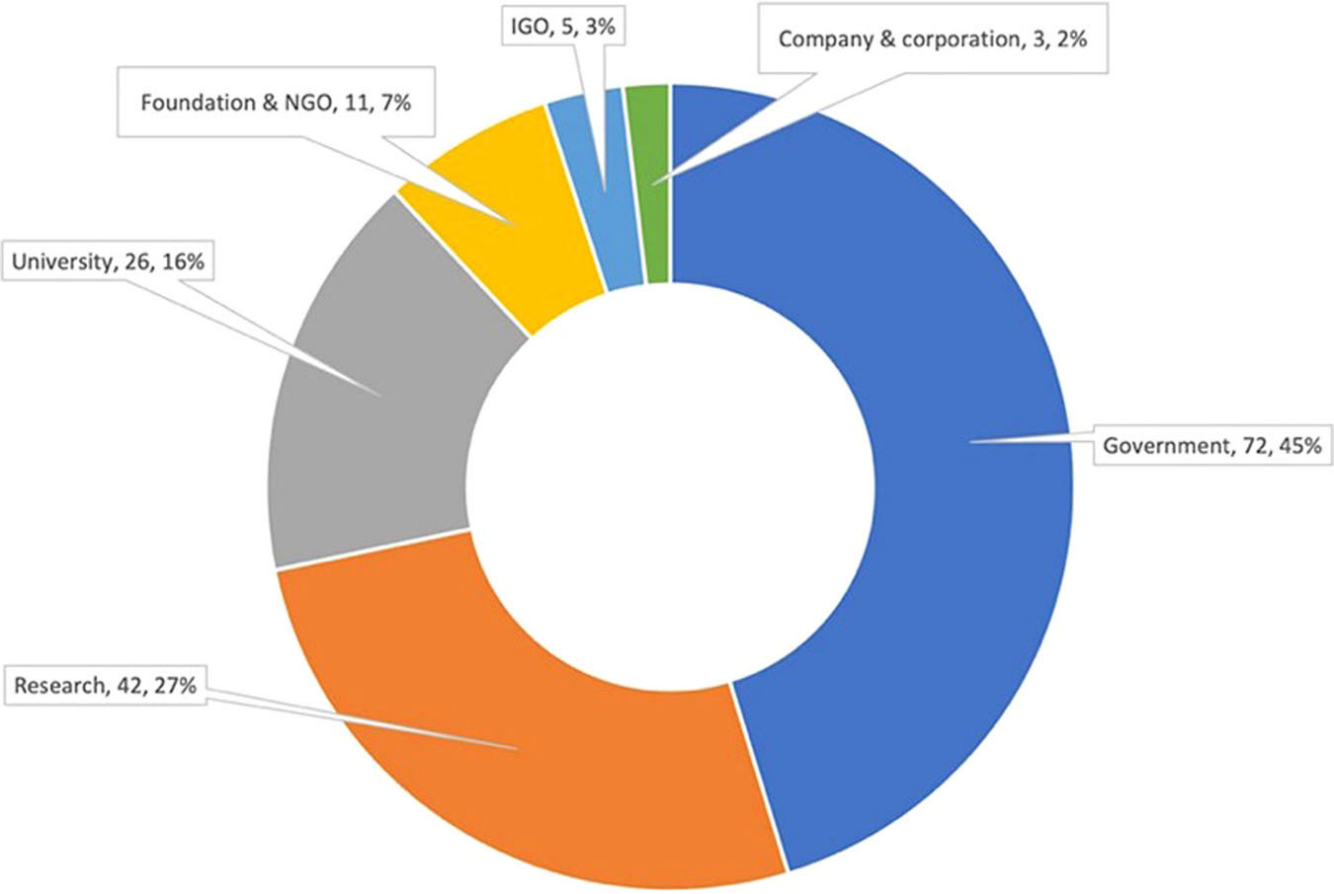
Types of funders of rice research in Indonesia and the Philippines since 2001, *n* = 159 funders. The majority of rice research projects in Indonesia and the Philippines were funded by governments (45%), research (27%), and university institutions (16%).

Only around 47% (1050 studies) of our dataset acknowledged their funding sources, where most received financial support either from governments (45%), research (27%), or university funders (16%) (Fig. 8). To see the top 15 funders that supported at least 10 rice-related research projects in Indonesia and the Philippines from 2001 to 2021, refer to Supplementary Table 5. Of over 150 rice research funders, Indonesia’s Ministry of Education, Culture, and Research (formerly the Ministry of Research and Technology) funded ~6% (62 out of 1050 studies). The Japan Society for the Promotion of Science and Japan’s Ministry of Education, Culture, Sports, Science and Technology came in as the second and third largest funders, respectively.

### Articles

Half of all articles in the dataset were borne out of interdisciplinary collaboration. More than a quarter of the articles, however, were unidentified, showing an apparent undercount of the total number of disciplinary collaborations. Most of these collaborative pieces of work (~61%) belong to the natural science subject areas of agricultural and biological sciences; biochemistry, genetics, and molecular biology; and environmental science (see Table 2). Note that the cumulative number of articles in Table 2 is more than the total number of the sample dataset since an article may belong to multiple subject areas as indicated by its authors in Scopus. Less than 9% (354) of all papers were written by social scientists, highlighting their marginal contribution to rice research. The social studies of rice can increase our understanding of the many facets of rice production, including their socio-political, economic, and cultural aspects.

**Table 2:** Top 10 subject areas of rice research in Indonesia and the Philippines since 2001.

Subject areas	Articles	Subject areas	Articles
Agricultural and Biological Sciences	1375	Economics, Econometrics, and Finance	137
Biochemistry, Genetics, and Molecular Biology	462	Earth and Planetary Sciences	129
Environmental Science	399	Medicine	112
Social Sciences	354	Computer Science	111
Engineering	209	Arts and Humanities	88

**Fig. 9:**
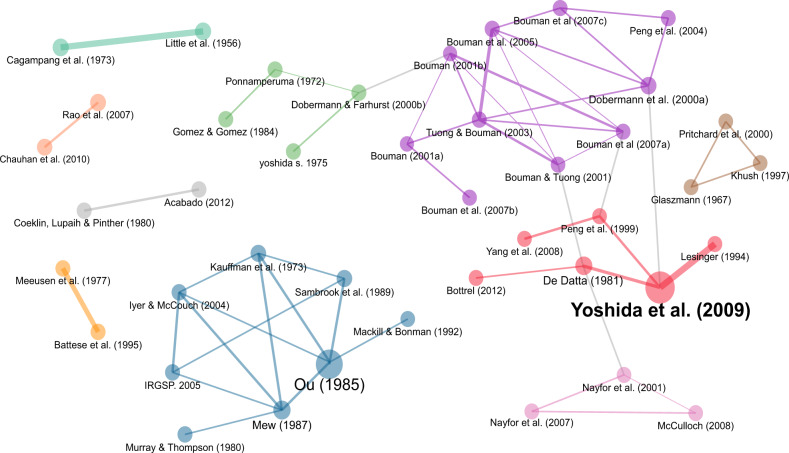
Article co-citation networks of rice research in Indonesia and the Philippines. There are 10 major networks of rice research co-citations in Indonesia and the Philippines.

Our review shows that there are 10 major networks of rice research co-citations (Fig. 9). The papers by Bouman et al. (2005), Bouman et al. (2007), Bouman and Tuong (2001), and Tuong and Bouman (2003) were co-cited by scholars studying the relationship between water scarcity management vis-à-vis rice growth and yield (the purple cluster in Fig. 9). Papers by Yoshida et al. (2009), De Datta (1981), and Peng et al. (1999) were co-cited by scholars researching the genetic diversity, yield, and principles and practices of rice production in Indonesia (the red cluster in Fig. 9). Papers by Ou (1985), Mackill and Bonman (1992), Sambrook et al. (1989), Kauffman et al. (1973), Iyer and McCouch (2004), and Mew (1987) were considered essential references in studying rice diseases (blue cluster in Fig. 9). The top-cited article on rice research in Indonesia and the Philippines, based on their overall global citations, is a study on water-efficient and water-saving irrigation (Belder et al., 2004). This study detailed alternative options for typical water management in lowland rice cultivation, where fields are continuously submerged, hence requiring a continuous large amount of water supply (Belder et al., 2004). Global citations refer to the citations received by the articles within and beyond our sample dataset of 2243 articles. To see the top 10 most globally cited articles on rice research in Indonesia and the Philippines, refer to Supplementary Table 6.

### Journals

The journal *Biodiversitas:* Journal of Biological Diversity published the most number of papers on rice research in the two countries. *Biodiversitas* publishes papers “dealing with all biodiversity aspects of plants, animals, and microbes at the level of gene, species, ecosystem, and ethnobiology” (Biodiversitas, 2021). Following its indexing in Scopus in 2014, *Biodiversitas* has increasingly published rice studies, most of which were authored by Indonesian researchers. To see the top 10 most relevant journals for rice research in Indonesia and the Philippines based on the number of documents published since 2001, refer to Supplementary Table 7.

Based on their local citations, the journals *Field Crops Research*, *Theoretical & Applied Genetics*, and *Science* are the most relevant. *Field Crops Research* focuses on crop ecology, crop physiology, and agronomy of field crops for food, fiber, feed, medicine, and biofuel. *Theoretical and Applied Genetics* publishes original research and review articles in all critical areas of modern plant genetics, plant genomics, and plant biotechnology. *Science* is the peer-reviewed academic journal of the American Association for the Advancement of Science and one of the world’s top academic journals. To see the top 30 most relevant journals for rice research in Indonesia and the Philippines based on the number of local citations, refer to Supplementary Table 8.

### Knowledge hotspots

**Fig. 10:**
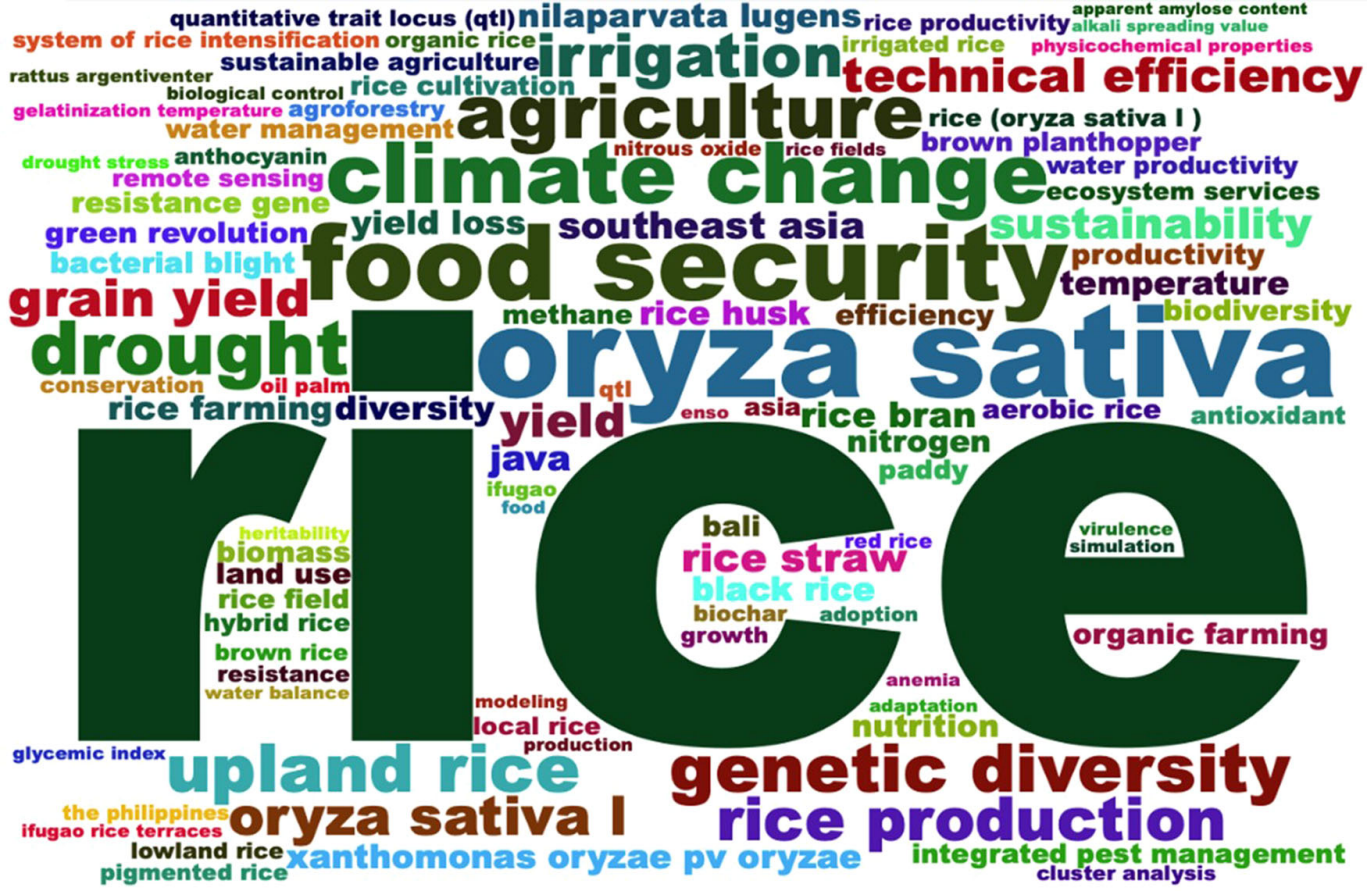
Top 100 keywords on rice research in Indonesia and the Philippines since 2001, *n* = 6482 authors’ keywords. The most used keywords found in 2243 rice research papers published between 2001 and 2021 in Indonesia and the Philippines are food security, climate change, drought, agriculture, irrigation, genetic diversity, sustainability, technical efficiency, and production.

**Fig. 11:**
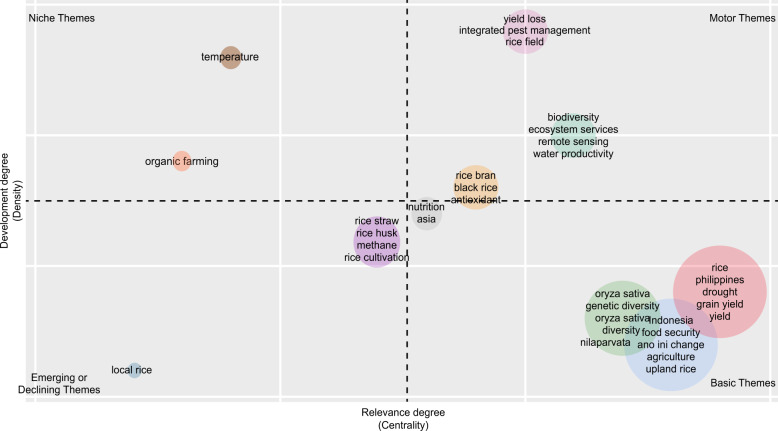
Knowledge Hotspots of rice research in Indonesia and the Philippines since 2001. There are four major themes composed of 11 clusters of rice research in Indonesia and the Philippines since 2001.

The most used keywords found in 2243 rice research papers published between 2001 and 2021 in Indonesia and the Philippines are food security, climate change, drought, agriculture, irrigation, genetic diversity, sustainability, technical efficiency, and production (Fig. 10). We found 11 clusters across four significant themes of rice research in these countries (Fig. 11).

#### Basic themes

We identified four major clusters under ‘basic themes’ (refer to Fig. 11):

- The Red Cluster on studies in the Philippines related to rice yield and productivity, drought, nitrogen, the Green Revolution, and the use and potential of biomass;
- The Blue Cluster on studies in Indonesia related to food security, climate change, agriculture, upland rice, irrigation, technical efficiency, and sustainability vis-à-vis rice production;
- The Green Cluster on rice genetic diversity, bacterial blight diseases, resistant rice genes, aerobic rice, and brown planthoppers; and
- The Gray Cluster on the nutritional aspects of rice, including studies on biofortified rice cultivars.

Agriculture suffers from climate change impacts and weather extremes. Rice researchers in Indonesia and the Philippines are identifying drought-tolerant rice cultivars that can produce high yields in abiotic stress-prone environments (Afa et al., 2018; Niones et al., 2021). These hybrid cultivars are vital for increasing rice productivity, meeting production demand, and feeding the growing Filipino and Indonesian populations (Kumar et al., 2021; Lapuz et al., 2019). Researchers have also looked at alternative nutrient and water management strategies that farmers can use, especially those in rainfed lowland areas during drought (Banayo, Bueno et al., 2018; Banayo, Haefele et al., 2018). There were also studies on the socio-cultural dynamics under which farmers adapt to droughts, such as how past experiences of hazards influence farmers’ perceptions of and actions toward drought (Manalo et al., 2020).

#### Motor themes

We identified three significant clusters of ‘motor themes’ (refer to Fig. 11):

- The Pink Cluster on yield loss and integrated pest management of rice fields;
- The Blue-Green Cluster on biodiversity, ecosystem services, remote sensing, and water productivity; and
- The Orange Cluster on the antioxidant properties of rice bran and black rice.

In both countries, pests, including weeds (Awan et al., 2014, 2015), insects (Horgan et al., 2018, 2021), and rodents (Singleton, 2011; Singleton et al., 2005, 2010), have significant impacts on yield loss in rice production and human health. To address these, many farmers have embraced chemical-heavy pest management practices to prevent yield loss and increase economic benefits. Pesticides began their use in Indonesia and the Philippines and rapidly expanded from the 1970s to the 1980s (Resosudarmo, 2012; Templeton and Jamora, 2010). However, indiscriminate use of pesticides caused an ecological imbalance that exacerbated pest problems (Templeton and Jamora, 2010) and contributed to farmers’ acute and chronic health risks (Antle and Pingali, 1994; Pingali and Roger, 1995).

Integrated pest management was introduced, applied, and studied in both countries to address these issues. This approach combines multiple compatible pest control strategies to protect crops, reduce pesticide use, and decrease farming costs (Gott and Coyle, 2019). For example, Indonesia’s 1989 National Integrated Pest Management Program trained hundreds of thousands of farmers and agricultural officials about its principles, techniques, and strategies (Resosudarmo, 2012). In the Philippines, the government of then-President Fidel V. Ramos (1992–1996) prohibited using hazardous pesticides and instituted a “multi-pronged approach to the judicious use of pesticides” (Templeton and Jamora, 2010, p. 1). President Ramos’ suite of policies included deploying Integrated Pest Management “as a national program to encourage a more ecologically sound approach to pest control” (Templeton and Jamora, 2010, p. 1). This pesticide policy package benefited the Philippine government in terms of private health costs avoided (Templeton and Jamora, 2010).

To address weed problems, farmers traditionally use manual weeding, a labor-intensive practice. However, as labor costs for manual weeding increased, herbicide use became economically attractive to farmers (Beltran et al., 2012). Herbicide experiments were made to address common rice weeds including barnyard grass (*Echinochloa crus-galli*) (Juliano et al., 2010), crowfoot grass (*Dactyloctenium aegyptium*) (Chauhan, 2011), three-lobe morning glory (*Ipomoea triloba*) (Chauhan and Abugho, 2012), and jungle rice (*Echinochloa colona*) (Chauhan and Johnson, 2009). Knowledge gained from these experiments contributed to the development of integrated weed management strategies.

Yet, many factors come into play when farmers decide to use herbicides. Beltran et al. (2013) reported that farmers’ age, household size, and irrigation use are significant determinants of adopting herbicides as an alternative to manual weeding. Beltran et al. (2013) further showed that economic variables, like the price of the herbicide, household income, and access to credit, determined farmers’ level of herbicide use (Beltran et al., 2013). Their study highlights the complex decision-making process and competing factors affecting weed management in the Philippines.

Apart from weeds, insects, like brown planthoppers (*Nilaparvata lugens*) and green leafhoppers (*Cicadella viridis*) and their accompanying diseases, affect rice production. In Java, Indonesia, Triwidodo (2020) reported a significant influence between the insecticide use scheme and the brown planthopper (*Nilaparvata lugens*) attack rates in rice fields. Brown planthopper attacks increased depending on the frequency of pesticide application, their varieties, and volume (Triwidodo, 2020). In the Philippines, Kim and colleagues (2019) developed a rice tungro epidemiological model for a seasonal disaster risk management approach to insect infestation.

Some social studies of integrated pest management included those that looked at the cultural practices that mitigate insect pest losses (Litsinger et al., 2011) and farmers’ knowledge, attitudes, and methods to manage rodent populations (Stuart et al., 2011). Other social scientists evaluated the value of amphibians as pest controls, bio-monitors for pest-related health outcomes, and local food and income sources (Propper et al., 2020).

#### Niche themes

We identified two ‘niche themes’ consisting of studies related to (a) temperature change and (b) organic rice production (refer to Fig. 11). Temperature change significantly affects rice farming. In the Philippines, Stuecker et al. (2018) found that El Niño-induced soil moisture variations negatively affected rice production from 1987–2016. According to one experiment, high night temperature stress also affect rice yield and metabolic profiles (Schaarschmidt et al., 2020). In Indonesia, a study suggests that introducing additional elements, such as Azolla, fish, and ducks, into the rice farming system may enhance rice farmers’ capacity to adapt to climate change (Khumairoh et al., 2018). Another study produced a rainfall model for Malang Regency using Spatial Vector Autoregression. This model is essential as rainfall pattern largely determines the cropping pattern of rice and other crops in Indonesia (Sumarminingsih, 2021).

Studies on organic rice farming in the Philippines include resource-poor farmers’ transition from technological to ecological rice farming (Carpenter, 2003) and the benefits of organic agriculture in rice agroecosystems (Mendoza, 2004). Other studies on organic rice focused on its impacts on agricultural development (Broad and Cavanagh, 2012) and climate resilience (Heckelman et al., 2018). In Indonesia, Martawijaya and Montgomery (2004) found that the local demand for organic rice produced in East Java was insufficient to generate revenue enough to cover its production costs. In West Java, Komatsuzaki and Syuaib (2010) found that organic rice farming fields have higher soil carbon storage capacity than fields where rice is grown conventionally. In Bali, farmers found it challenging to adopt organic rice farming vis-à-vis the complex and often contradictory and contested administration of the Subaks (MacRae and Arthawiguna, 2011) and the challenges they have to confront in marketing their produce (Macrae, 2011).

#### Emerging or declining themes

We identified two clusters of ‘emerging/declining themes’ or areas of rice research that are weakly developed and marginal (refer to Fig. 11). The Purple Cluster (emerging) studies rice straw, rice husk, methane, and rice cultivation, while the Light Blue Cluster (declining) pertains to local rice research.

## Contributions from and research agenda for the social sciences

In this section, we present and discuss the contributions of the social sciences, highlight key gaps, and provide a research agenda across six interdisciplinary areas for future studies. In Table 3, we summarized the various topic areas that other scholars could focus on in their future studies of rice in Indonesia and the Philippines.

**Table 3:** A summary of potentially novel research topics across six interdisciplinary subject areas of rice research in Indonesia and the Philippines.

Interdisciplinary subject areas	Future works
Economic, political, and policy studies	Comparative studies on agricultural land-use change, land management, urbanization, food and agricultural policies, trade policies, irrigation governance, and price dynamics.
Sociological, anthropological, and cultural studies	Temporal and spatial studies on the social, cultural, technological, and human–ecological interactions of different rice farming regions in Indonesia and the Philippines.
Social and environmental psychology	Research on sociopsychological processes such as attitude and behavior, social cognition, self and identity, individual differences, emotions, human–environmental health and well-being, social influence, and cultural processes. Psycho-behavioral areas of nature–people interactions, theories of place, place attachment, and place identity, especially in rice farming.
Climate change, weather extremes, and disaster risk reduction	Studies on climate information and environmental monitoring; nature-based solutions for climate mitigation and adaptation; water–energy–food nexus in rice farming; the nexus of climate change and conflict in rice farming communities; just energy transitions in rice farming; and local-indigenous strategies on adapting to climate change.
Demographic transitions and aging farmers	Studies on aging’s potential negative implications in terms of agricultural efficiency and productivity, food security, farming continuity and sustainability, aging and feminization of farm labor, cleaner production behaviors, youth barriers to farm entry, and health and well-being of aging farmers. (Dis)engagement and re-engagement of young people in rice farming; gender dynamics—including structures and systems of inclusion and/or exclusion—in rice production; the impacts of migration and return migration to farming households; community-based and policy-oriented case studies that provide examples of successfully engaging and retaining youth workers in farming; and social protection measures for aging farmers.
Contemporary and emerging challenges	Studies on the Covid-19 impacts on the vulnerabilities of rice farmers and other rice value chain actors, specifically its medium- and longer-term effects on rice production, unemployment risks, rice supply and nutrition security of farming households, and the potential and extent to which economic stimulus can benefit rice farmers.

### Economic, political, and policy studies

Political scientist Ernest A. Engelbert (1953) was one of the earliest scholars to summarize the importance of studying agricultural economics, politics, and policies. Engelbert (1953) identified three primary reasons scholars and laypeople alike need to understand the nature of political processes in agriculture. First, the rapid change and highly contested political environment where agriculture operates often places agriculture last on national policy agenda. Second, the formulation of agricultural policies intersects with contemporary national and economic contexts by which these policies revolve. Third, understanding the political processes around agriculture can help avoid political pressures and machinations aimed at undermining agricultural development.

Politics play a crucial role in better understanding rice- and agriculture-related policies, their evolution, dynamics, challenges, developments, and futures. Grant (2012, p. 271) aptly asks, “Who benefits [from government policies, regulations, and programs]?” . Knowing, understanding, and answering this question is crucial since policymaking is a highly contested process influenced and negotiated not only by farmers and decision-makers but also by other interest groups, such as people’s organizations and non-government organizations. On the other hand, understanding macro- and micro-economic government arrangements come hand-in-hand in analyzing how policies impact farmers and consumers. Using tariffs as an example, Laiprakobsup (2014, p. 381) noted the effects of government interventions in the agrarian market:

“… when the government implements consumer subsidy programs by requiring the farmers to sell their commodities at a cheaper price, it transfers the farmers’ incomes that they were supposed to earn to the consumers. Moreover, the government transfers tax burdens to the farmers via export taxes in that the agricultural industry is likely to purchase the farmers’ commodities as cheaply as possible in order to make up for its cost.”

The two countries have compelling economic, political, and policy-oriented rice studies. Some examples of this type of research in the Philippines are the following. Intal and Garcia (2005) argued that the price of rice had been a significant determinant in election results since the 1950s. Fang (2016) analyzed how the Philippines’ colonial history bolstered an oligarchy system, where landed elite politicians and patronage politics perpetuated corruption to the detriment of rice farmers. Balié and Valera (2020) examined rice trade policy reforms’ domestic and international impacts. San Juan (2021) contends that the 2019 Rice Tariffication Law of the Philippines only encouraged the country to rely on imports and failed to make the local rice industry more competitive.

In Indonesia, some political studies on rice production are the following. Putra et al. (2020) analyzed how urbanization affected food consumption, food composition, and farming performance. Noviar et al. (2020) provided evidence that households in the rice sub-sector have achieved an insufficient level of commercialization in their rice production. Rustiadi et al. (2021) investigated the impacts of land incursions over traditionally rice farming regions due to Jakarta’s continuous expansion. Satriawan and Shrestha (2018) evaluated how Indonesian households participated in the Raskin program, a nationwide rice price subsidy scheme for the poor. Misdawita et al. (2019) formulated a social accounting matrix and used a microsimulation approach to assess the impacts of food prices on the Indonesian economy.

#### Future work

Social science researchers could further explore and compare the local, regional, and national similarities and differences of the abovementioned issues or conduct novel research related to land-use change, land management, urbanization, food and agricultural policies, trade policies, irrigation governance, and price dynamics. Comparative social studies of rice could also lead to meaningful results. As social policy scholar Linda Hantrais noted:

“Comparisons can lead to fresh, exciting insights and a deeper understanding of issues that are of central concern in different countries. They can lead to the identification of gaps in knowledge and may point to possible directions that could be followed and about which the researcher may not previously have been aware. They may also help to sharpen the focus of analysis of the subject under study by suggesting new perspectives.” (Hantrais, 1995, p. n/a).

### Sociological, anthropological, and cultural studies

Biologists dominated agricultural research until the mid-1960s (Doorman, 1991). Agriculture, in other words, was no social scientist’s business. However, this situation gradually changed when governments and scholars realized the long-term impacts of the Green Revolution from the 1950s to the 1980s, which underscores that the development, transfer, and adoption of new agrotechnology, especially in developing countries, is driven not only by techno-biological factors but also by the socio-economic, political, and cultural realities under which the farmers operate. Since then, sociologists, anthropologists, and cultural scholars have become indispensable in answering the “how”, “what”, and “why” agrarian communities follow, adopt, utilize, or, in some cases, prefer local/traditional production technologies over the technological and scientific innovations developed by engineers, biologists, geneticists, and agriculturists. Nyle C. Brady, a soil scientist and the former Director-General of the IRRI pointed out:

“… we increasingly recognize that factors relating directly to the farmer, his family, and his community must be considered if the full effects of agricultural research are to be realized. This recognition has come partly from the participation of anthropologists and other social scientists in interdisciplinary teams … during the past few years.” (IRRI, 1982).

Since the late 19th century, many rice studies have tried to answer the roles of social scientists in agricultural research. Social sciences have contributed to agricultural research in many ways, especially regarding technology adoption by farmers (DeWalt, 1985; Doorman, 1990). Doorman (1991, p. 4) synthesized these studies and offered seven roles for sociologists and anthropologists in agricultural research as follows:

“Accommodator of new technology, ex-post and ex-ante evaluator of the impact of new technology, an indicator of the needs for new technology, translator of farmer’s perceptions, broker-sensitizer, adviser in on-farm research, and trainer of team members from other disciplines.”

Social studies of rice are especially critical in Indonesia and the Philippines—home to hundreds of Indigenous cultural communities and Indigenous peoples (Asian Development Bank, 2002; UNDP Philippines, 2010). Regardless of the highly contested debates surrounding “indigeneity” or “being indigenous,” especially in Indonesia (Hadiprayitno, 2017), we argue that Indigenous cultural communities and Indigenous peoples have similarities (i.e., they are often farming or agrarian societies) but also recognize their differences and diversity in terms of their farming practices, beliefs, traditions, and rituals. These socio-cultural factors and human and non-human interactions influence rice production; thus, these differences and diversity bring front-and-center the importance of needs-based, community-driven, and context-sensitive interventions or projects for rice farming communities. These are research areas best explored by sociologists, anthropologists, and cultural scholars.

Today, agriculture’s sociological, anthropological, and cultural research have gone beyond the classic technology adoption arena. In Indonesia, studies have explored farmers’ technical efficiency in rice production (e.g., Muhardi and Effendy, 2021), the similarities and differences of labor regimes among them (e.g., White and Wijaya, 2021), the role of social capital (e.g., Salman et al., 2021), and the reciprocal human–environmental interactions in the rice ecological system (e.g., Sanjatmiko, 2021). Disyacitta Nariswari and Lauder (2021) conducted a dialectological study to examine the various Sundanese, Javanese, and Betawi Malay words used in rice production. Rochman et al. (2021) looked into the *ngahuma* (planting rice in the fields) as one of the inviolable customary laws of the *Baduy* Indigenous cultural community in Banten, Indonesia.

In the Philippines, Balogbog and Gomez (2020) identified upland rice farmers’ productivity and technical efficiency in Sarangani. Aguilar et al. (2021) examined the drivers of change, resilience, and potential trajectories of traditional rice-based agroecosystems in Kiangan, Ifugao. Pasiona et al. (2021) found that using the “modified listening group method” enables farmers’ peer-to-peer learning of technical concepts. Sociologist Shunnan Chiang (2020) examined the driving forces behind the transformation of the status of brown rice in the country.

#### Future work

Social scientists could further look into the social, cultural, technological, and human–ecological interactions in the temporal and spatial studies of different rice farming regions in Indonesia and the Philippines. Other topics could include the cultural practices and the techno-social relationships of rice farmers (e.g., Shepherd and McWilliam, 2011) and other players in the rice value chain, local and indigenous knowledge and practices on agrobiodiversity conservation, historical and invasive pests and diseases, agricultural health and safety, farmer education, and aging agricultural infrastructures. Lastly, future researchers can explore the impacts of adopting rice farming technologies in the different stages or processes of the rice value chain. They can look into its short- and longer-term effects on farmers’ livelihoods and conduct comparative analyses on how it improves, or not, their livelihoods, and whether farmers regard them better compared to the traditional and indigenous practices and beliefs that their communities apply and observe in rice farming.

### Social and environmental psychology

Our review yielded no article published on the social and environmental psychology aspects of rice farming in Indonesia and the Philippines, suggesting a new research frontier. The increasing demand for and competition over agricultural and natural resources due to climate change and population expansion (Foley et al., 2011) opens up new and emerging sociopsychological dilemmas for society to understand, answer, and, hopefully, solve. Social and environmental psychologists can help shed light on these questions, such as those related to understanding farmers’ pro-environmental agricultural practices (Price and Leviston, 2014), sustainable sharing and management of agricultural and natural resources (Anderies et al., 2013; Biel and Gärling, 1995), and understanding the psychosocial consequences of resource scarcity (Griskevicius et al., 2013). Broadly, social psychology examines human feelings, thoughts, and behaviors and how they are influenced by the actual, imagined, and implied presence, such as the effects of internalized social norms (Allport, 1985). Social psychologists look at the many facets of personality and social interactions and explore the impacts of interpersonal and group relationships on human behavior (American Psychological Association, 2014b). On the other hand, environmental psychology examines psychological processes in human encounters with their natural and built environments (Stern, 2000). Environmental psychologists are interested in studying and understanding people’s responses to natural and technological hazards, conservation, and perceptions of the environment (American Psychological Association, 2014a).

#### Future work

Using the *Asian Journal of Social Psychology* and the *Journal of Environmental Psychology* as benchmarks, we recommend that scholars explore the following uncharted or least studied areas of rice research in Indonesia and the Philippines: sociopsychological processes such as attitude and behavior, social cognition, self and identity, individual differences, emotions, human–environmental health and well-being, social influence, communication, interpersonal behavior, intergroup relations, group processes, and cultural processes. Researchers could also investigate the psycho-behavioral areas of nature–people interactions, theories of place, place attachment, and place identity, especially in rice farming. Other topics may include farmers’ perceptions, behaviors, and management of environmental risks and hazards; theories of pro-environmental behaviors; psychology of sustainable agriculture; and the psychological aspects of resource/land management and land-use change.

### Climate change, weather extremes, and disaster risk reduction

Indonesia’s and Philippines’ equatorial and archipelagic location in the Pacific Ring of Fire (Bankoff, 2016; Parwanto and Oyama, 2014), coupled with their political, social, and economic complexities (Bankoff, 2003, 2007; UNDRR and CRED, 2020), expose and render these countries highly vulnerable to hazards, such as typhoons, strong winds, tsunamis, storm surges, floods, droughts, and earthquakes. The accelerating global climate change increases the frequency and intensity of some of these hazards, such as prolonged droughts, torrential rainfalls causing floods, and super typhoons (IPCC, 2014). For example, torrential flooding, induced by heavy rains caused by low pressures and southwest monsoons, has been damaging lives and livelihoods, including rice production (Statista, 2021). The 2020 droughts caused over 12 trillion pesos (~US$239.40 billion) of economic losses in the Philippines (Statista, 2021) and affected millions of Indonesians (UNDRR, 2020). Prolonged drought in Indonesia has also exacerbated fire hazards, which caused transboundary haze pollution in neighboring countries, like Singapore and the Philippines, inflecting environmental health damages (Aiken, 2004; Sheldon and Sankaran, 2017; Tan-Soo and Pattanayak, 2019). Increasing sea-level rise due to anthropogenic climate change puts cities like Jakarta and Manila at risk of sinking in the next 30–50 years (Kulp and Strauss, 2019). The high vulnerability, frequent exposure, and low capacities of marginalized and poor Indonesians and Filipinos turn these hazards into disasters (Gaillard, 2010; Kelman, 2020; Kelman et al., 2015), negatively affecting rice agriculture.

Given these contexts, climate change, weather extremes, and disaster risks, vis-à-vis its impacts on the rice sector, are issues of profound interest to scholars and the Indonesian and Philippine governments. In the Philippines, climate adaptation studies include re-engineering rice drying systems for climate change (Orge et al., 2020) and evaluating climate-smart farming practices and the effectiveness of Climate-Resiliency Field Schools in Mindanao (Chandra et al., 2017). In Indonesia, where some rice farming communities are vulnerable to sea-level rise, scholars are experimenting to identify rice cultivars with high yields under different salinity levels (Sembiring et al., 2020). Hohl et al. (2021) used a regional climate model to develop index-based drought insurance products to help the Central Java government make drought-related insurance payments to rice farmers. Aprizal et al. (2021) utilized land-use conditions and rain variability data to develop a flood inundation area model for the Way Sekampung sub-watershed in Lampung, Sumatra. Others also looked at the science behind liquefaction hazards caused by irrigation systems for wet rice cultivation in mountainous farming communities like the 2018 earthquake-triggered landslides in Palu Valley, Sulawesi (Bradley et al., 2019).

Examples of climate mitigation-related studies in the Philippines include investigating the social innovation strategies in engaging rice farmers in bioenergy development (Minas et al., 2020) and evaluating the environmental performance and energy efficiency of rice straw-generated electricity sources (Reaño et al., 2021). Doliente and Samsatli (2021) argue that it is possible to combine energy and food production to increase farm productivity and reduce GHG emissions with minimal land expansion. Other studies have looked into the potential of alternate wetting and drying irrigation practices to mitigate emissions from rice fields (Sander et al., 2020).

#### Future work

Future work could explore the following topic areas: demand-driven research and capacity building on climate information and environmental monitoring; nature-based solutions for climate mitigation and adaptation; water–energy–food nexus in rice farming; the nexus of climate change and conflict in rice farming communities; the potentials and pitfalls of social capital in farmer’s everyday adaptation; just energy transitions in rice farming; vulnerabilities from and traditional/local/indigenous ways of adapting to climate change, including the various learning strategies communities use for its preservation; and examples, potentials, and barriers in adopting climate-smart agriculture technologies and practices.

### Demographic transitions and aging farmers

Farmers are in various stages and speeds of aging globally (Rigg et al., 2020). Evidence of aging farmers in the Global North has been reported in Australia (O’Callaghan and Warburton, 2017; Rogers et al., 2013), the Czech Republic (Zagata et al., 2015), England (Hamilton et al., 2015), Japan (Poungchompu et al., 2012; Usman et al., 2021), and the United States of America (Mitchell et al., 2008; Reed, 2008; Yudelman and Kealy, 2000). Similarly, in the Global South, HelpAge International (2014, p. 21) reported that “there has been a universal trend of an increase in the proportion of older people… attached to agricultural holdings… across [Low and Middle-income Countries in] Asia, sub-Saharan Africa, Latin America, and the Caribbean.” Moreover, farming populations are aging rapidly in East and Southeast Asia (Rigg et al., 2020) and southern Africa (HelpAge, 2014). Despite this, the literature on aging farmers in Southeast Asian countries remains scant, except for case studies conducted in some villages and provinces in Thailand (Poungchompu et al., 2012; Rigg et al., 2018, 2020) and the Philippines (Moya et al., 2015; Palis, 2020).

#### Future work

Rice farmers’ quiet but critical demographic transformation in Indonesia and the Philippines has not received much attention from scientists, policymakers, and development practitioners. The impacts of aging farmers on the micro-, meso-, and macro-level agricultural processes and outcomes are important issues that require urgent attention. Studies done in other countries could guide future work to explore these questions in Indonesia and the Philippines. These include aging’s potential negative implications in terms of agricultural efficiency and productivity (e.g., Tram and McPherson (2016) in Vietnam, and Szabo et al. (2021) in Thailand), food security (e.g., Bhandari and Mishra (2018) in Asia), farming continuity and sustainability (e.g., O’Callaghan and Warburton (2017) in Australia, Palis (2020) in the Philippines, and Rigg et al. (2018, 2020) in Thailand), aging and feminization of farm labor (e.g., Liu et al. (2019) in China), cleaner production behaviors (e.g., Liu et al. (2021) in Northern China), youth barriers to farm entry (e.g., Zagata and Sutherland (2015) in Europe), and health and well-being of aging farmers (Jacka, 2018; Rogers et al., 2013; Ye et al., 2017).

Other critical new topics include the (dis)engagement and re-engagement of young people in rice farming; gender dynamics—including structures and systems of inclusion and/or exclusion—in rice production; the impacts of migration and return migration to farming households; community-based and policy-oriented case studies that provide examples of successfully engaging and retaining youth workers in farming; and social protection measures for aging farmers, to name a few.

### Contemporary and emerging challenges

One of the biggest and most visible contemporary global challenges is the Covid-19 pandemic. Most pronounced is the pandemic’s impacts on the healthcare system and the economic toll it caused on the lives and livelihoods of people, including rice farmers. Only 0.18% (4 articles) of our dataset have investigated the impacts of Covid-19 on rice systems in Indonesia and the Philippines. Ling et al. (2021) assessed the effects of the pandemic on the domestic rice supply vis-à-vis food security among ASEAN member-states. They found that Singapore and Malaysia were highly vulnerable to a pandemic-induced rice crisis, while Brunei, Indonesia, and the Philippines are moderately vulnerable. They argued that Southeast Asian rice importers should consider alternative import strategies to reduce their high-risk reliance on rice supply from Thailand and Vietnam and look for other suppliers in other continents.

Rice prices did not change in the early months of the pandemic in Indonesia (Nasir et al., 2021); however, as the health emergency progressed, distributors and wholesalers incurred additional costs due to pandemic-induced mobility restrictions (Erlina and Elbaar, 2021). In the Philippines, San Juan (2021) argues that the global rice supply disruption due to the pandemic proves that the country cannot heavily rely on rice imports; instead, it should work on strengthening its domestic rice supply. To realize this, he recommended drastic investments in agriculture and research, rural solar electrification, and the promotion of research on increasing rice yields, boosting productivity, and planting sustainably as feasible steps on the road to rice self-sufficiency.

#### Future work

The ways and extent to which the pandemic negatively affected or exacerbated the vulnerabilities of rice farmers and other value chain actors remain an understudied area in the social studies of rice. Scholars could study the pandemic’s impacts in conjunction with other contemporary and emerging challenges like climate change, weather extremes, aging, conflict, and poverty. Scholars could also explore the medium- and longer-term impacts of the pandemic on rice production, unemployment risks, rice supply and nutrition security of farming households, and the potential and extent to which economic stimulus can benefit rice farmers, to name a few. Most importantly, the pandemic allows researchers and governments to assess the business-as-usual approach that resulted in the disastrous impacts of the pandemic on different sectors, including rice farmers, and hopefully devise strategies to learn from these experiences.

## Conclusion

From our review of 2243 articles, cumulatively written by 6893 authors using almost 80,000 references, we conclude that a voluminous amount of rice research has been conducted in Indonesia and the Philippines since 2001. As in other reviews, (e.g., on energy research by Sovacool, 2014), our results show that women scholars remain underrepresented in rice research in Indonesia and the Philippines. While interdisciplinary collaboration is abundant, most of these studies belong to the natural sciences with minimal contributions from the social sciences, arts, and humanities. University and research institutions contributed the most to rice research in Indonesia and the Philippines: from hybrid rice cultivars, water management, and technology adoption to socio-cultural, political, economic, and policy issues. Influential scholars in the field were affiliated with the IRRI, which can be expected given the institute’s focus on rice, and key agriculture-focused universities and government bureaus such as the University of the Philippines and the PhilRice in the Philippines, and the Institut Pertanian Bogor University and the Universitas Gadja Maja in Indonesia. We also discussed some examples of economic, political, and policy studies; social, anthropological, and cultural research; social and environmental psychology; climate change, weather extremes, and disaster risk reduction; demographic transitions; and contemporary and emerging issues and studies on rice in the two Southeast Asian countries. Ultimately, we hope that this systematic review can help illuminate key topic areas of rice research in Indonesia and the Philippines and magnify the crucial contributions from and possible research areas and questions that interdisciplinary and comparative social scientists can further explore.

## Supplementary information

Supplementary Info

## Data Availability

The dataset analyzed in this study is available in the Figshare online repository via 10.6084/m9.figshare.17284814.v2. All codes about Bibliometrix are available at https://bibliometrix.org/.
