# Supplementary material for: Two decades of rice research in Indonesia and the Philippines: A systematic review and research agenda for the social sciences
Source: Humanit Soc Sci Commun. 2022 Oct 14;9(1):372. doi: 10.1057/s41599-022-01394-z (PMC9562066; doi:10.1057/s41599-022-01394-z)
Supplement: Supplementary file 1 — Supplementary Info [file 41599_2022_1394_MOESM1_ESM.docx]

SUPPLEMENTARY FILE for **Two decades of rice research in Indonesia and the Philippines: a systematic review and research agenda for the social sciences**

**Authors**

Ginbert P. Cuaton^1^ and Laurence L. Delina^1^

**Affiliation**

^1^ The Hong Kong University of Science and Technology, Hong Kong SAR, China

**Corresponding Author**

Ginbert P. Cuaton, [gcuaton@ust.hk](mailto:gcuaton@ust.hk)

**Supplementary Table 1. Authors with at least ten articles on rice research in Indonesia and/or the Philippines, n= 37**

| **Authors** | **Articles** | **Authors** | **Articles** |
| --- | --- | --- | --- |
| Horgan, F. G. | 21 | Canapi, B. L. | 11 |
| Bouman, B. A. M. | 20 | Khush, G. S. | 11 |
| Singleton, G. R. | 20 | Kobayashi, N. | 11 |
| Fukuta, Y. | 19 | Stuart, A. M. | 11 |
| Chauhan, B. S. | 17 | Angeles-Agdeppa, I. | 10 |
| Kumar, A. | 16 | Aswidinnoor, H. | 10 |
| Litsinger, J. A. | 16 | Barrion, A. T. | 10 |
| Peng, S. | 16 | Capanzana, M. V. | 10 |
| Wassmann, R. | 15 | Dingkuhn, M. | 10 |
| Iskandar, J. | 14 | Heong, K. L. | 10 |
| Almazan, M. L. P. | 13 | Johnson, D. E. | 10 |
| Herlinda, S. | 13 | Lakitan, B. | 10 |
| Kato, Y. | 13 | Purwoko, B. S. | 10 |
| Partasasmita, R. | 13 | Ramal, A. F. | 10 |
| Settele, J. | 13 | Suwarno, W. B. | 10 |
| Juliano, B. O. | 12 | Telebanco-Yanoria, M. J. | 10 |
| Bernal, C. C. | 11 | Vera Cruz, C. M. | 10 |

**Supplementary Table 2. Most locally cited authors, n= 20**

| **Author** | **Citations** | **Author** | **Citations** |
| --- | --- | --- | --- |
| Iskandar, J. | 36 | Corpuz, D. C. P. | 19 |
| Muhidin, M. | 30 | Rahni, N. M. | 19 |
| Partasasmita, R. | 28 | Sutariati, G. A. K. | 19 |
| Madiki, A. | 25 | Wibawa, G. | 19 |
| Abdullah, L. | 22 | Fukuta, Y. | 18 |
| Chandra, R. | 22 | Bueno, C. S. | 17 |
| Despal, D. | 22 | Clerget B. | 17 |
| Permana, I. G. | 22 | Sukono, S. | 17 |
| Sari, L. A. | 22 | Daulae, A. H. | 16 |
| Zahera, R. | 22 | Hasairin, A. | 16 |

**Supplementary Table 3. Top 10 rice scholars with the highest H-indices**

| **Author** | **H-index** | **Author** | **H-index** |
| --- | --- | --- | --- |
| Bouman, B. A. M. | 18 | Chauhan, B. S. | 11 |
| Fukuta, Y. | 13 | Kumar, A. | 11 |
| Peng, S. | 13 | Almazan, M. L. P. | 10 |
| Horgan, F. G. | 12 | Settele, J. | 10 |
| Wassmann, R. | 12 | Singleton, G. R. | 10 |

**Supplementary Table 4. Rice research productivity, according to the authors’ country of publication, n= 20 countries**

| **Country** | **Articles** | **Country** | **Articles** |
| --- | --- | --- | --- |
| Indonesia | 2024 | The United Kingdom | 86 |
| The Philippines | 1149 | France | 72 |
| Japan | 477 | Thailand | 57 |
| United States of America | 448 | Republic of South Korea | 55 |
| Republic of China | 242 | Italy | 34 |
| Australia | 241 | New Zealand | 33 |
| Germany | 220 | Canada | 29 |
| Malaysia | 110 | Bangladesh | 27 |
| The Netherlands | 107 | Switzerland | 26 |
| India | 96 | Belgium | 20 |

**Supplementary Table 5. Top 15 funders of rice research in Indonesia and the Philippines**

| **Funder** | **Number of studies funded** |
| --- | --- |
| Ministry of Education, Culture, and Research (Indonesia) | 62 |
| Japan Society for the Promotion of Science | 43 |
| Ministry of Education, Culture, Sports, Science, and Technology (Japan) | 33 |
| Australian Centre for International Agricultural Research | 31 |
| Universitas Gadjah Mada (Indonesia) | 26 |
| Bill and Melinda Gates Foundation (USA) | 25 |
| National Science Foundation (USA) | 24 |
| National Natural Science Foundation of China | 23 |
| Department of Foreign Affairs and Trade (Australia) | 22 |
| The Swiss Agency for Development and Cooperation | 17 |
| Agricultural Research Development Agency (Thailand) | 16 |
| Federal Ministry of Education and Research (Germany) | 16 |
| The Consortium of International Agricultural Research Centers | 16 |
| Universitas Padjadjaran (Indonesia) | 15 |
| German Research Foundation | 14 |

**Supplementary Table 6. Top 10 rice research in Indonesia and the Philippines with the most number of global citations.**

| **Author & Date** | **Title** | **Journal** | **DOI** | **Citation** |
| --- | --- | --- | --- | --- |
| Belder et al. (2004) | “Effect of water-saving irrigation on rice yield and water use in typical lowland conditions in Asia” | Agricultural Water Management | 10.1016/j.agwat.2003.09.002 | 438 |
| Bouman et al. (2005) | “Yield and water use of irrigated tropical aerobic rice systems” | Agricultural Water Management | 10.1016/j.agwat.2004.11.007 | 396 |
| Septiningsih et al. (2009) | “Development of submergence-tolerant rice cultivars: The Sub1 locus and beyond.” | Annals of Botany | 10.1093/aob/mcn206 | 319 |
| Christian et al. (2003) | “Comprehensive laboratory measurements of biomass-burning emissions: Emissions from Indonesian, African, and other fuels.” | Journal of Geophysical Research: Atmospheres | 10.1029/2003jd003704 | 298 |
| Tabbal et al. (2002) | “On-farm strategies for reducing water input in irrigated rice: Case studies in the Philippines.” | Agricultural Water Management | 10.1016/S0378-3774(02)00007-0 | 288 |
| Frei et al. (2003) | “Studies on the in vitro starch digestibility and the glycemic index of six different indigenous rice cultivars from the Philippines.” | Food Chemistry | 10.1016/S0308-8146(03)00101-8 | 261 |
| Krerkkaiwan et al. (2013) | “Synergetic effect during co-pyrolysis/gasification of biomass and sub-bituminous coal” | Fuel Processing Technology | 10.1016/j.fuproc.2013.03.044 | 239 |
| Naylor et al. (2007) | “Assessing risks of climate variability and climate change for Indonesian rice agriculture.” | PNAS of the USA | 10.1073/pnas.0701825104 | 191 |
| Haas et al. (2005) | “Iron-biofortified rice improves the iron stores of non-anemic Filipino women.” | Journal of Nutrition | 10.1093/jn/135.12.2823 | 167 |
| Nishimura et al. (2006) | “Decrease in polyamines with aging and their ingestion from food and drink.” | Journal of Biochemistry | 10.1093/jb/mvj003 | 162 |

*Note: Global citations refer to the citations the listed articles received from articles within and beyond our sample dataset of 2,243 articles.*

**Supplementary Table 7. Most relevant rice research journals, based on the number of documents published, 2001-2021**

| **Journal** | **Publications** |
| --- | --- |
| Biodiversitas | 104 |
| Philippine Agricultural Scientist | 48 |
| Field Crops Research | 44 |
| International Journal of Advanced Science Engineering | 39 |
| Journal of the International Society for Southeast Asian Agricultural Sciences | 27 |
| Philippine Journal of Science | 26 |
| Agrivita | 22 |
| Crop Protection | 17 |
| Sabrao Journal of Breeding and Genetics | 17 |
| Soil Science and Plant Nutrition | 16 |

**Supplementary Table 8. Most rice research-relevant journals based on local citations, n= 30.**

| **Journal** | **Publisher** | **Local Citations** |
| --- | --- | --- |
| Field Crops Research | Elsevier Ltd | 1011 |
| Theoretical & Applied Genetics | Springer | 577 |
| Science | American Association for the Advancement of Science | 362 |
| Nature | Springer Nature | 356 |
| Crop Science | John Wiley & Sans Ltd on behalf of the American Society of Agronomy, Crop Science Society of America, and Soil Science Society of America | 337 |
| Phytopathology | The American Phytopathological Society | 315 |
| PLOS One | Public Library of Science | 302 |
| Plant and Soil | Springer | 294 |
| Euphytica | Springer | 236 |
| Genetics | Oxford University Press | 231 |
| Plant Physiology | Oxford University Press on behalf of the American Society of Plant Biologists | 229 |
| Applied & Environmental Microbiology | American Society of Microbiology | 222 |
| Biodiversitas Journal of Biological Diversity | Biology Department of Sebelas Maret University | 214 |
| Agronomy Journal | John Wiley & Sans Ltd on behalf of the American Society of Agronomy | 198 |
| Food Policy | Elsevier Ltd | 198 |
| Rice | Springer Open | 191 |
| Proceedings of the National Academy of Sciences of the USA | United States of America National Academy of Sciences | 169 |
| Plant Cell | Oxford University Press on behalf of the American Society of Plant Biologists | 167 |
| Geoderma | Elsevier | 163 |
| Soil Science Society of America Journal | John Wiley & Sans Ltd on behalf of the Soil Science Society of America | 161 |
| World Development | Elsevier Ltd | 156 |
| Soil Biology and Biochemistry | Elsevier Ltd | 151 |
| Journal of Experimental Botany | Oxford University Press on behalf of the Society for Experimental Biology | 150 |
| Plant Journal | John Wiley & Sans Ltd in association with the Society for Experimental Biology | 150 |
| Agriculture, Ecosystems, & Environment | Elsevier Ltd | 149 |
| Journal of Geophysical Research | John Wiley & Sans Ltd on behalf of the American Geophysical Union | 147 |
| American Journal of Clinical Nutrition | Oxford University Press on behalf of the American Society for Nutrition | 142 |
| Bulletin of Indonesian Economic Studies | Taylor & Francis Ltd on behalf of the ANU Indonesia Project | 141 |
| Agricultural Systems | Elsevier Ltd | 139 |
| Plant Production Science | Taylor & Francis Ltd on behalf of the Crop Science Society of Japan | 139 |

*Note: Local citations refer to the citations received by the listed articles from the sample dataset of 2,243 articles.*
